# Genomic structural equation modeling elucidates the genetic mechanisms underlying allostatic load

**DOI:** 10.1016/j.cpnec.2026.100357

**Published:** 2026-06-25

**Authors:** Songhao Chai, Wenjun Jin, Zhenghao Cui, Bo Li

**Affiliations:** a The First Affiliated Hospital of Zhengzhou University, Zhengzhou, Henan, 450001, China; b Medical Department, SIAS University, Zhengzhou, Henan, 451150, China

**Keywords:** Allostatic load, Genomic SEM, Post-GWAS, TWAS, HNF4A, Chronic stress biology

## Abstract

**Background:**

Allostatic load (AL) represents the cumulative physiological burden arising from chronic stress across neuroendocrine, immune, metabolic, and cardiovascular systems. Although AL is strongly associated with cardiometabolic and inflammatory diseases, its underlying genetic architecture remains poorly characterized.

**Methods:**

We integrated genome-wide association summary statistics for five AL-related phenotypes—systolic blood pressure, white blood cell count, C-reactive protein, body mass index, and triglycerides—using Genomic Structural Equation Modeling (Genomic SEM) to derive a latent genetic factor indexing the shared cardiovascular, inflammatory, and metabolic components of AL. A common-factor model was fitted to derive a latent genetic representation of AL, followed by multivariate GWAS of the latent factor. Downstream analyses included functional annotation, Bayesian fine-mapping, transcriptome-wide association analysis (TWAS), pathway enrichment, cell type–specific heritability estimation, and partitioned SNP heritability analyses.

**Results:**

The common-factor model showed good fit (CFI = 0.964; SRMR = 0.037), supporting a shared genetic architecture across the five AL-related traits. Functional annotation indicated that AL-associated variants were predominantly located in non-coding regulatory regions. Pathway enrichment analyses implicated metabolic regulation, lipid processing, neuroendocrine signaling, and immune-related pathways. Integrative fine-mapping and TWAS prioritized genes involved in metabolic homeostasis and neuronal signaling, including HNF4A, MLXIPL, BDNF, and SH2B1. Cell type–specific analyses showed enrichment in neuronal populations and stress-related brain regions, while partitioned heritability analyses demonstrated significant enrichment in conserved regions, promoters, enhancers, and active chromatin marks.

**Conclusion:**

This study characterizes the shared polygenic architecture of an AL-related latent factor derived from cardiovascular, inflammatory, and metabolic biomarkers. The results suggest that genetic liability captured by this AL-related latent factor converges on metabolic, immune-inflammatory, neuroendocrine and neural regulatory pathways, providing a system-level perspective on AL-related physiological burden and multisystem disease vulnerability.

## Highlights

• Genomic SEM identified a latent genetic factor capturing multisystem allostatic load.
• Genetic liability to allostatic load reflects coordinated metabolic and neurobiological regulation.
• Integrative fine-mapping and TWAS prioritized metabolic and neuronal genes, including HNF4A, MLXIPL, BDNF, and SH2B1.
• Cell-type and chromatin analyses implicated stress-related brain regions and regulatory elements.

## 1 Introduction

In modern society, individuals are continuously exposed to multiple environmental, psychological, and social stressors, such as high occupational demands, socioeconomic disparities, and unhealthy lifestyle patterns. These chronic stressors interact through complex neuroendocrine, immune, metabolic, and cardiovascular regulatory pathways, imposing cumulative physiological burdens on the body. Over time, such sustained regulatory strain can compromise the organism's ability to maintain internal stability, leading to multisystem dysfunction—a state conceptualized as Allostatic Load (AL) [1]. As a conceptual framework linking psychosocial stress to disease susceptibility, AL provides a means to understand how chronic stress becomes biologically embedded and contributes to adverse health outcomes.

From a biological perspective, the stress response is a dynamic adaptive process designed to restore homeostasis in the face of environmental challenges. Activation of the hypothalamic–pituitary–adrenal (HPA) axis and sympathetic nervous system mobilizes energy reserves [2], elevates cardiovascular output, and temporarily enhances immune vigilance. However, when these responses become frequent or prolonged, chronic neuroendocrine activation may result in systemic dysregulation, including sustained inflammation, impaired insulin signaling, persistent hypertension, oxidative stress, and immune dysfunction [3,4]. These interconnected pathophysiological processes constitute key biological pathways through which AL contributes to long-term cardiovascular, metabolic, and neural vulnerability.

Extensive empirical evidence supports strong associations between elevated AL and a wide range of adverse health outcomes, including cardiovascular disease, metabolic disorders, cognitive decline, and increased mortality [[5], [6], [7]]. Among these outcomes, the cardiovascular and metabolic systems appear particularly sensitive to chronic stress–related regulatory overload. Elevated AL has been linked to endothelial dysfunction, atherosclerotic progression, insulin resistance, and increased risk of cardiometabolic disease [8]. In parallel, sustained inflammatory activation associated with high AL has been implicated in immune dysregulation and heightened vulnerability to chronic disease progression [9]. Despite this well-established epidemiological evidence, the genetic architecture and molecular pathways that confer individual susceptibility to elevated AL remain poorly understood, motivating the need for integrative genomic investigation.

Existing genetic evidence directly related to AL remains limited and fragmented, with most prior studies relying on candidate-gene approaches. Previous work has reported associations between AL and polymorphisms in genes involved in cardiovascular regulation, neuroendocrine stress response, and serotonergic signaling, including ACE, CRHR1, HTR3A, and HTR4, although only the ACE polymorphism rs4968591 remained significant after correction for multiple comparisons [10]. Variants in the APOA1/C3/A4/A5 gene cluster have also been examined in relation to AL markers, supporting a potential contribution of lipid-metabolism genes to AL-related physiological burden [11]. Similarly, genetic variation in glucocorticoid receptor signaling has been associated with AL in adolescent populations [12]. Nevertheless, these studies have generally focused on selected candidate genes or predefined pathways and have not characterized the genome-wide shared genetic architecture of AL as a complex multisystem construct. Therefore, a multivariate genome-wide framework is needed to move beyond isolated candidate mechanisms and to systematically prioritize genes and pathways associated with AL-related physiological burden.

The advent of large-scale genome-wide association studies (GWAS) has provided unprecedented opportunities to investigate the genetic basis of complex traits. However, conventional univariate GWAS approaches are limited in their ability to capture the shared genetic architecture underlying correlated physiological systems involved in AL. Genomic Structural Equation Modeling (Genomic SEM) offers a powerful approach to model the shared and specific genetic components of complex traits by estimating genetic covariance structures based solely on GWAS summary statistics [13]. This framework accounts for sample overlap and enables latent genetic factors to be derived from correlated traits, making it suitable for investigating higher-order constructs such as AL. Its application to psychiatric, metabolic, and cardiovascular phenotypes further supports its utility for studying complex multisystem traits and syndrome-like constructs [14,15].

Building on this framework, the present study extends the genetic analysis of AL from single-biomarker associations to a latent, system-level representation of shared physiological burden. We model the genetic covariance among cardiovascular, inflammatory, and metabolic indicators as an AL-related latent construct and examine whether this shared genetic component converges on biologically interpretable pathways, regulatory contexts, and tissue or cell-type signatures. This framing emphasizes AL-related physiological dysregulation as a coordinated multisystem liability rather than a collection of isolated biomarkers.

## 2 Methods

### 2.1 Sources of univariate GWAS data

To construct a genetic representation of AL, we constructed a latent genetic factor from five biomarkers representing three major physiological domains commonly used in empirical AL indices: cardiovascular regulation, systemic inflammation, and metabolic burden. These included systolic blood pressure (SBP) as a cardiovascular indicator, white blood cell count (WBC) and C-reactive protein (CRP) as immune-inflammatory indicators, and body mass index (BMI) and triglycerides (TG) as metabolic burden.

In this framework, AL was not modeled as a directly observed clinical phenotype, but as a latent genetic construct capturing the shared genetic covariance across these selected AL-related physiological domains. This operational definition is consistent with the broader conceptualization of AL as cumulative multisystem physiological dysregulation, while allowing the construct to be estimated using large-scale harmonized GWAS summary statistics.

All GWAS summary statistics were obtained exclusively from the IEU OpenGWAS database, which hosts harmonized summary-level data from large population-based cohorts and international consortia (https://gwas.mrcieu.ac.uk/). To reduce potential population stratification, we included only GWAS datasets conducted in individuals of European ancestry. Detailed information for each contributing GWAS, including phenotype definition, GWAS accession number, ancestry composition, sex, sample size, data source, and OpenGWAS phenotype description, is provided in Supplementary Table 1. Each contributing GWAS had received ethical approval from the appropriate institutional review boards, and written informed consent had been obtained from all participants in the original studies.

**Fig. 1:**
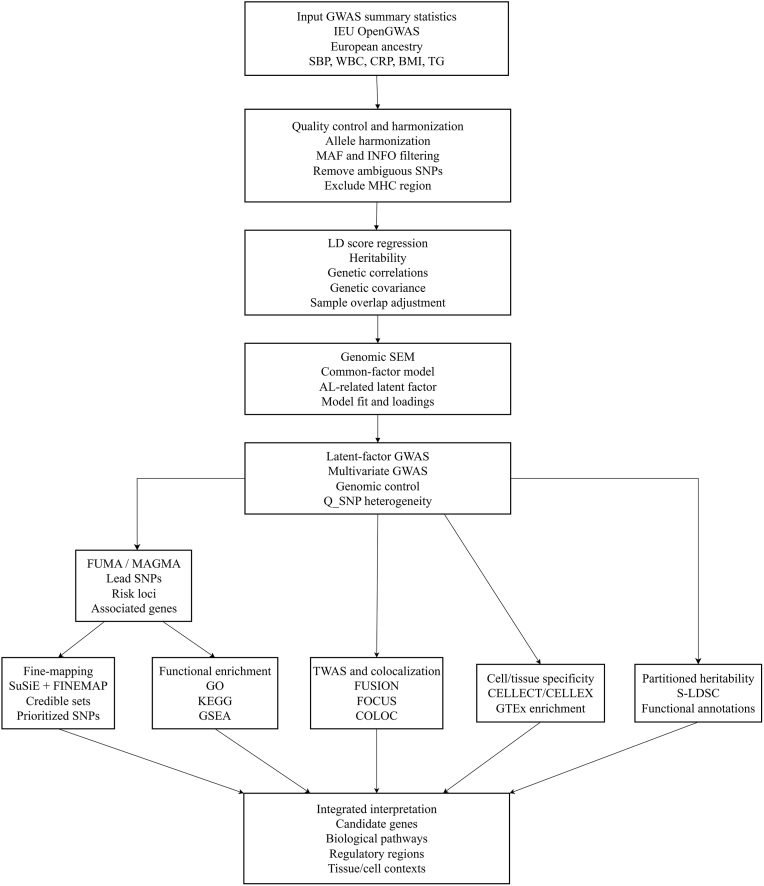
Step-by-step analytical workflow for genetic modeling of AL The workflow summarizes the study pipeline from GWAS summary statistic selection, quality control, LD score regression, and Genomic SEM to latent-factor GWAS and downstream post-GWAS analyses, including FUMA/MAGMA annotation, fine-mapping, functional enrichment, TWAS, cell/tissue specificity analysis, and partitioned heritability analysis.

The overall analytical workflow, from GWAS selection and quality control to Genomic SEM, latent-factor GWAS, and downstream post-GWAS analyses, is summarized in Fig. 1.

### 2.2 Ethical considerations

This study was conducted entirely on publicly available genome-wide association study (GWAS) summary statistics; no additional ethics approval was required. All original GWAS had prior approval from their respective institutional review boards and obtained informed consent from participants. No individual-level genetic or clinical data were accessed.

### 2.3 Quality control of univariate GWAS

To ensure cross-study comparability and analytical reliability, all summary statistics underwent a unified quality-control and harmonization pipeline:

1. Variant Filtering: Using the 1000 Genomes Phase 3 (EUR) reference, variants with minor allele frequency (MAF) < 0.01, imputation INFO <0.9, or allele mismatches were removed.
2. Ambiguous SNPs: Palindromic A/T and G/C SNPs were excluded to avoid strand ambiguity.
3. The Major Histocompatibility Complex (MHC) region: The extended MHC (chr6: 25–35 Mb) was excluded owing to extensive linkage disequilibrium and complex haplotype structure.
4. Sample overlap adjustment: Where input GWAS potentially shared participants (e.g., partial UK Biobank overlap), cross-trait LD Score regression intercepts were used to adjust for residual sample correlation [16].

### 2.4 Sample overlap in univariate GWAS

In our analysis, the included univariate GWASs were derived from different genomic data repositories, each with distinct participant cohorts. This means that we carefully accounted for potential sample overlap across cohorts to ensure the accuracy and generalizability of our results, while also addressing the possible statistical implications associated with overlapping samples.

### 2.5 Genomic structural equation modeling

To characterize the shared genetic architecture underlying multiple AL–related traits, we applied Genomic SEM, a multivariate framework that estimates latent genetic factors from GWAS summary statistics while accounting for genetic correlations and sample overlap across traits. Because AL represents a latent construct reflecting multisystem physiological dysregulation rather than a single directly observable phenotype, a univariate GWAS approach is insufficient to capture its shared genetic basis,we used Genomic SEM to estimate the shared genetic component across the selected cardiovascular, inflammatory, and metabolic indicators. Genomic SEM (R package v0.0.5) therefore provides an appropriate framework to quantify the common genetic liability underlying its component traits while separating shared from trait-specific genetic effects [13]. Genomic SEM is a multivariate extension of LD score regression that enables the estimation of latent genetic factors underlying multiple complex traits using genome-wide summary statistics.

To appropriately account for potential sample overlap and imbalanced sample sizes across GWAS, we relied on the cross-trait LD score regression framework to estimate the genetic variance–covariance matrix, including cross-trait intercepts that adjust for shared samples. This overlap-adjusted covariance matrix served as the input for all subsequent Genomic SEM analyses.

The Genomic SEM analysis was conducted in two stages. First, multivariate LD score regression was used to estimate SNP-based heritabilities and pairwise genetic covariances across the five phenotypes. Second, a single common-factor structural equation model was specified and fitted to the genetic covariance matrix to derive a latent genetic factor indexing AL, while allowing for trait-specific residual genetic effects.

Model specification and fit: Model fit was assessed using several indices: Chi-square statistic (χ^2^): Evaluates the degree of fit between the hypothesized model and the observed data [17]; Comparative Fit Index (CFI): Compares the fit of the target model with a null model in which all variables are uncorrelated; Akaike Information Criterion (AIC): Used to compare the relative quality of different models, lower AIC values indicate better model fit [18]; Standardized Root Mean Square Residual (SRMR): Measures the discrepancy between the predicted and observed covariance matrices. We defined good model fit using the following thresholds: CFI ≥0.95 and SRMR <0.08 [14].

### 2.6 SNP heterogeneity testing and multi-level evaluation

Although AL is conceptualized as a shared latent construct, individual genetic variants may exert effects that are not fully mediated through the common factor. The Q_SNP statistic allows formal testing of such heterogeneity at the SNP level. To test whether individual SNPs exert effects beyond the latent common factor, we calculated the Q_SNP statistic within the multivariate framework. Under the null, univariate SNP associations are fully mediated through the latent AL factor [19]. Therefore, a significant Q_SNP statistic (P < 0.05) indicates that the SNP exerts additional effects not captured by the shared genetic architecture of AL, reflecting potential trait-specific, system-specific, or pleiotropic influences.

### 2.7 Locus identification and functional annotation

While Genomic SEM identifies genome-wide loci associated with the latent AL factor, additional functional annotation is required to map these signals onto genes and biological pathways. We employed the Functional Mapping and Annotation of Genetic Associations (FUMA) platform to perform functional mapping and annotation of genomic loci associated with the latent allostatic factor. This approach identifies key genomic regions and highlights lead SNPs that meet the genome-wide significance threshold (P < 5 × 10^−8^) and show low linkage disequilibrium (r^2^ < 0.1) with nearby variants. Summary statistics from the Genomic SEM analysis were uploaded to FUMA to evaluate SNP–trait associations, and the resulting lead SNPs were compared with those from previously reported univariate GWAS to examine concordance and potential novelty [20].

Based on the FUMA analysis, a risk locus analysis was conducted using the same genome-wide significance threshold to identify additional loci associated with the allostatic factor. To further explore gene-level associations, we applied Multi-marker Analysis of GenoMic Annotation (MAGMA) to aggregate multiple SNP signals into gene-based statistics and evaluate the relationship between individual genes and the latent factor [21]. The significance threshold used in this analysis was an FDR-adjusted P-value <0.05.

Finally, we compared lead SNPs obtained from the Genomic SEM model with those from univariate GWAS analyses of the individual component traits. This comparison allowed us to identify novel and functionally relevant loci that were specific to the shared genetic structure underlying AL, thereby providing additional insights into its polygenic architecture.

### 2.8 Fine-mapping analysis

To identify the most likely causal variants associated with AL, we applied two widely used Bayesian fine-mapping methods: Sum of Single Effects (SuSiE) [22] and FINEMAP [23], implemented through the R package (echolocatoR v2.0.3).

First, fine-mapping analyses were performed using both SuSiE and FINEMAP to pinpoint variants with high probabilities of being causal for the phenotype. For each lead SNP, a ±250 kb window around the index variant was defined to capture the surrounding genomic region, and the posterior inclusion probability (PIP) was computed for each SNP within this interval.

A posterior probability threshold of 0.95 was applied to construct credible sets, representing collections of variants with the highest probability of containing the true causal variant. Variants exceeding this threshold were considered putative causal SNPs with strong statistical support.

Using the echolocatoR framework, we further identified consensus SNPs, defined as variants consistently included in the credible sets derived from both SuSiE and FINEMAP. For these consensus SNPs, the tool calculated the mean posterior probability and defined a mean credible set. Variants with posterior probabilities greater than 0.95 in both methods were assigned a confidence score of 1 in the mean credible set, whereas all others were assigned a score of 0.

### 2.9 Transcriptome-wide association studies (TWAS)

Because most GWAS variants reside in non-coding regions, gene expression mediation represents a key mechanism linking genetic variation to physiological phenotypes. TWAS was therefore used to prioritize genes whose genetically regulated expression may mediate AL-related associations. To prioritize genes whose expression may mediate AL-related genetic associations, we performed TWAS using the FUSION framework with GTEx v8 eQTL weights across 49 tissues. Predicted expression–trait associations were tested, and genes meeting FDR <0.05 were considered significant and were cross-referenced with fine-mapping results to highlight candidates supported by both expression and causality evidence [24].

### 2.10 Functional enrichment and pathway analysis

Gene-level association results from the Genomic SEM GWAS were obtained using MAGMA as implemented in FUMA. Autosomal genes were retained, and gene-level P values were corrected for multiple testing using the false discovery rate (FDR). Genes with FDR <0.01 were considered significantly associated with the latent AL factor.

The resulting set of 2313 significant genes was subjected to Gene Ontology (GO) and Kyoto Encyclopedia of Genes and Genomes (KEGG) enrichment analyses using the *clusterProfiler* R package [25]. Pathway enrichment significance was evaluated using Benjamini–Hochberg FDR correction (adjusted P < 0.05).

### 2.11 Gene set and disease enrichment analysis

We conducted gene set enrichment and pathway analysis on AL-associated genes using the GSEA framework implemented in clusterProfiler [26]. The purpose of this step was to identify biological pathways and curated functional gene sets that show significant over-representation of AL-linked genes after multiple-testing correction. Enriched pathways were interpreted as potential molecular systems underlying chronic stress-related physiological dysregulation.

### 2.12 Cell type specificity analysis

Identifying the specific cell types through which AL-associated genetic variation acts is essential for understanding its biological implementation. We therefore performed cell type–specific heritability enrichment using single-cell transcriptomic data. To identify the etiological cell types potentially contributing to AL, we applied the CELL-type Expression-specific integration for Complex Traits (CELLECT) framework, which integrates single-cell RNA sequencing (scRNA-seq) data with complex trait summary statistics [27]. The analysis utilized the Tabula Muris dataset, which includes transcriptomic profiles of approximately 100,000 cells derived from 20 organs and tissues of *Mus musculus* [28].

Single-cell RNA sequencing data from Tabula Muris were preprocessed and normalized using CELLEX, which estimates the expression specificity likelihood score (ESμ) for each gene. These specificity scores were then integrated into LDSC regression to evaluate cell type–specific heritability enrichment, allowing systematic identification of cell populations genetically implicated in AL. Cell types were considered significantly enriched at an FDR <0.05.

### 2.13 Partitioned heritability analysis

To further characterize the regulatory architecture of AL-associated genetic variation, we conducted partitioned heritability analysis across functional genomic annotations. We performed partitioned SNP heritability analysis using Stratified Linkage Disequilibrium Score Regression (S-LDSC) to quantify the contribution of different functional genomic annotations to the total SNP-based heritability of the latent allostatic factor. This approach decomposes overall heritability into components attributable to specific genomic features, such as coding regions, conserved elements, transcription factor binding sites, and regulatory domains.

Functional annotation files and baseline models were obtained from the baselineLD v2.2 dataset provided by the LDSC resource. For each annotation category, enrichment was defined as the proportion of heritability explained divided by the proportion of SNPs within that category. Significance of enrichment was evaluated using regression coefficients and FDR–adjusted P values [16].

## 3 Result

### 3.1 Univariate GWAS characteristics and SNP-based heritability

We first examined the genomic characteristics of each phenotype included in the AL model using LD score regression. SNP-based heritability estimates (h^2^) were modest to moderate across traits, ranging from 0.125 for SBP to 0.248 for BMI. Detailed criteria for model construction are described in Table 1.

**Table 1:** LD Score regression of AL contributing phenotypes.

Allostatic Load component	Abbreviation	N SNPs	h2 (SE)	Lambda GC	Mean chi-square	Intercept (SE)	Ratio (SE)
Systolic blood pressure	SBP	1175041	0.1254 (0.005)	1.7665	2.1915	1.0977 (0.0174)	0.082 (0.0146)
White blood cell	WBC	1169557	0.1681 (0.0081)	1.7104	2.3624	1.1588 (0.0279)	0.1166 (0.0205)
C-reactive protein	CRP	1169349	0.138 (0.011)	1.6524	2.1132	1.1372 (0.048)	0.1233 (0.0431)
Body mass index (BMI)	BMI	1174996	0.2481 (0.0069)	2.4611	3.3686	1.1066 (0.022)	0.045 (0.0093)
Triglycerides	TG	1169265	0.1474 (0.0118)	1.6228	2.1958	1.1553 (0.0417)	0.1299 (0.0349)

### 3.2 Statistical indicators of structural equation modeling (SEM)

**Fig. 2:**
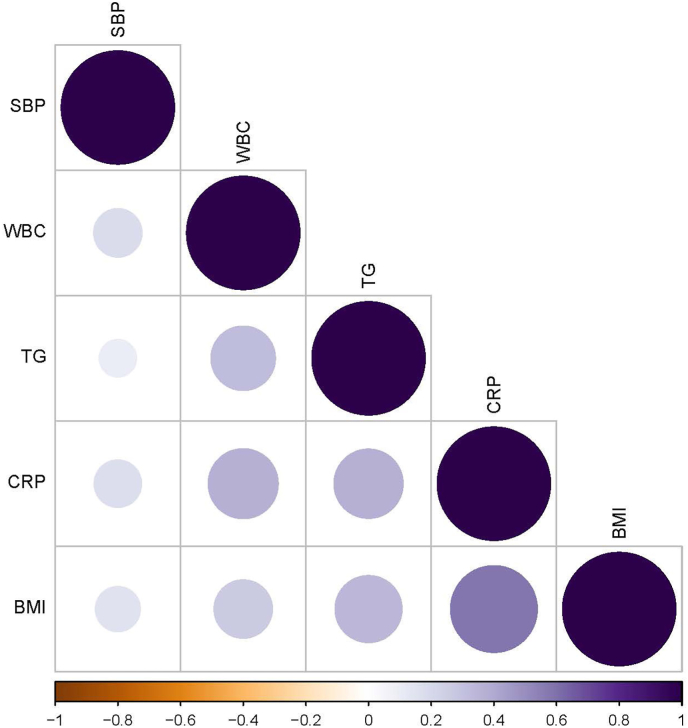
Genetic correlation structure among the five AL-related biomarkers used in the Genomic SEM model. Genetic correlations for SEM with genomic SEM, displaying pairwise LD score genetic correlation estimates for the 5 univariate phenotypes.

Pairwise genetic correlations among SBP, WBC, CRP, BMI, and TG were estimated using multivariate LD score regression (Single factor genetic covariance value detailed in Supplementary Table 2 and Fig. 2). All trait pairs showed significant positive genetic correlations, indicating substantial shared genetic architecture across cardiovascular, immune-inflammatory, and metabolic systems. Notably, stronger correlations were observed between immune-inflammatory and metabolic traits, including CRP–BMI (r_g = 0.583), CRP–TG (r_g = 0.370), and WBC–BMI (r_g = 0.263), whereas correlations involving SBP were comparatively more modest (r_g = 0.108–0.181). This pattern suggests that the latent factor captured a shared AL-related genetic component with relatively stronger contributions from inflammatory and metabolic indicators and a more modest contribution from the cardiovascular indicator.

Based on this genetic covariance structure, a single-factor Genomic SEM model was fitted to capture the shared latent genetic component across the selected AL-related indicators. The common-factor model demonstrated good overall fit to the data (CFI = 0.964, SRMR = 0.037; Supplementary Table 3), supporting the presence of a shared genetic component across these biomarkers. Examination of standardized factor loadings showed that CRP had the strongest loading on the latent factor (standardized loading = 0.839), followed by BMI (0.651), TG (0.532), WBC (0.466), and SBP (0.246). This loading pattern indicates that the latent factor was most strongly represented by inflammatory and metabolic indicators, with a more modest contribution from the cardiovascular indicator.

### 3.3 Genomic SEM GWAS evaluation and genomic control

Using the latent AL factor estimated through the Genomic SEM framework, we performed a genome-wide association analysis involving 7,111,933 SNPs. The quantile–quantile (Q–Q) plot showed a clear deviation from the null expectation, consistent with a polygenic genetic architecture of the latent AL factor (Supplementary Fig. 1).

To evaluate potential inflation and confounding, LD score regression was applied to the latent AL GWAS summary statistics after standard quality control and HapMap3 SNP filtering. The analysis yielded a mean chi-square statistic of 2.31 and a genomic inflation factor (λGC) of 1.90. The SNP-based heritability of the latent AL factor on the observed scale was low (h^2^ = 0.0011, SE = 2.99 × 10^−5^).

The LDSC intercept was close to unity (intercept = 0.96, SE = 0.016), indicating that the observed inflation was primarily driven by polygenic heritability rather than population stratification or other confounding effects. Overall, these results suggest that the latent AL GWAS contained a detectable polygenic signal with limited confounding, although the low observed-scale SNP heritability indicates that downstream gene- and pathway-level findings should be interpreted as prioritization signals rather than definitive mechanistic evidence.

### 3.4 FUMA-based evaluation of genomic SEM

**Fig. 3:**
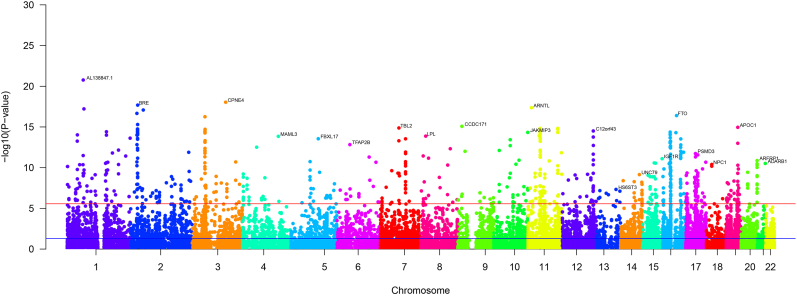
Manhattan plot of gene-based associations with the AL-related latent factor. The plot summarizes genome-wide gene-level association signals from FUMA analysis. The red line indicates the Bonferroni-adjusted significance threshold, highlighting loci prioritized for downstream functional interpretation.

Using FUMA, we identified 263 genomic risk loci and 2312 candidate genes associated with the latent allostatic factor under genome-wide significance criteria (FDR <0.01) (Supplementary Table 4,Fig. 3). Functional annotation of 410 lead SNPs identified by FUMA showed that the majority were located in non-coding regions. Specifically, 180 variants were intronic and 133 were intergenic, whereas 45 variants mapped to ncRNA-related regions and 18 were located in untranslated regions (UTRs). To further prioritize variants with potential functional impact, we focused on lead SNPs with high deleteriousness scores (CADD >10). Among these, both coding and regulatory variants were observed across genes involved in metabolic and neurobiological processes. For example, rs1800961, an exonic variant within the metabolic transcription factor gene HNF4A, showed a high CADD score (22.3), suggesting a potentially deleterious effect on metabolic regulation. In addition, rs6265 located within the BDNF locus (BDNF-AS:BDNF) was annotated as an exonic variant with a CADD score of 21.4, highlighting a possible role of neurobiological pathways in the genetic architecture of AL.

Overall, these findings indicate that the latent AL-related GWAS signals were predominantly located in non-coding regulatory regions, with selected coding variants providing candidate signals for metabolic and neurobiological processes.

### 3.5 KEGG pathway enrichment analysis

**Fig. 4:**
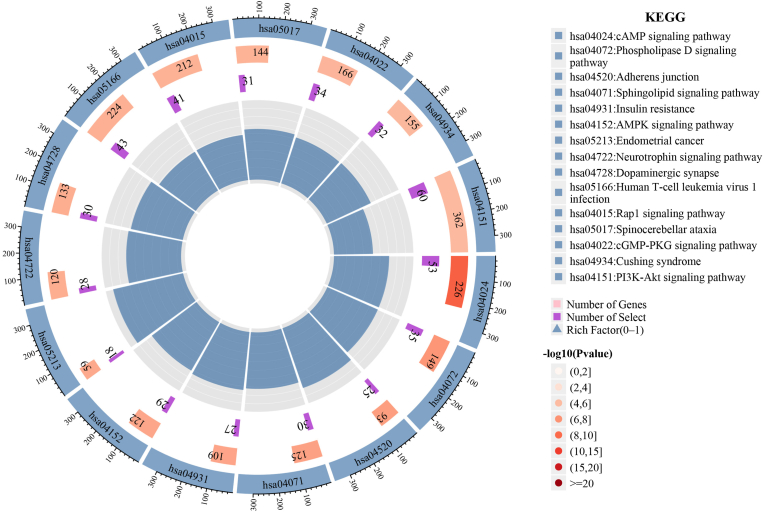
KEGG pathway enrichment of genes associated with the AL-related latent factor. The circular diagram shows the top enriched KEGG pathways identified from FUMA-based analysis. Each segment represents a pathway, with the outer ring indicating the KEGG ID, the middle blocks showing the number of background genes, and the inner arcs representing the count of genes enriched in each pathway. The color gradient corresponds to the –log_10_(P value). Enriched pathways were mainly related to metabolic signaling, endocrine regulation, neurobiological signaling, and immune-related processes. The circular plot displays the top enriched KEGG pathways, with color intensity corresponding to enrichment significance.

KEGG pathway analysis revealed that the genes associated with the latent allostatic factor were significantly enriched in multiple signaling and disease-related pathways (adjusted P < 0.05, Supplementary Table 5, Fig. 4). Among the most significantly enriched pathways were several core signal transduction cascades, including the cAMP signaling pathway (hsa04024), phospholipase D signaling pathway (hsa04072), sphingolipid signaling pathway (hsa04071), AMPK signaling pathway (hsa04152), and PI3K–Akt signaling pathway (hsa04151). These pathways are central regulators of cellular energy balance, stress sensing, and adaptive responses, suggesting that the shared genetic architecture of AL converges on broad intracellular signaling networks rather than isolated molecular targets.

Notably, pathways related to endocrine and metabolic dysfunction were also strongly enriched, including insulin resistance (hsa04931) and Cushing syndrome (hsa04934), highlighting the involvement of hormonal and metabolic dysregulation in the genetic basis of AL. This finding aligns with the conceptualization of AL as a cumulative burden arising from chronic stress–induced perturbations of metabolic and endocrine systems.

In addition, several nervous system–related pathways, such as neurotrophin signaling and dopaminergic synapse pathways, were significantly enriched, indicating that central nervous system signaling may contribute to the regulation of multisystem stress responses. Enrichment of immune- and infection-related pathways, including Human T-cell leukemia virus 1 infection, further suggests a role of chronic immune activation and inflammatory signaling in shaping the genetic architecture underlying AL.

### 3.6 GO pathway enrichment analysis

**Fig. 5:**
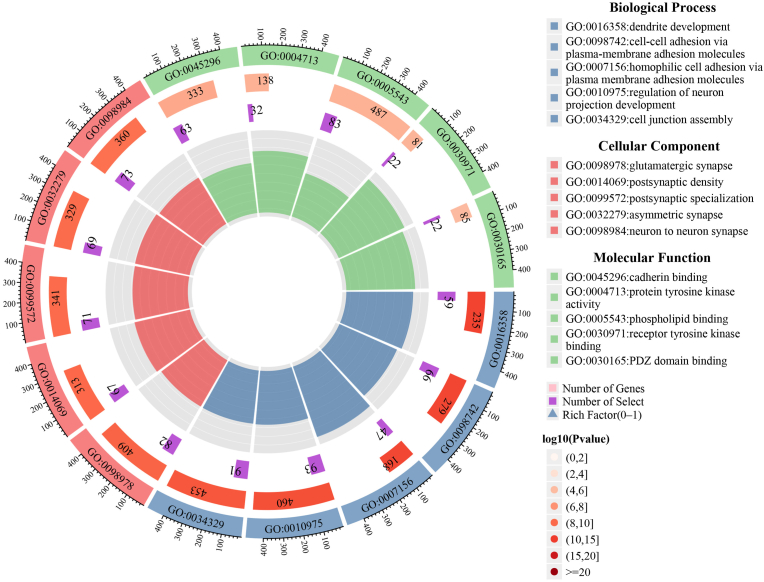
GO enrichment of genes associated with the AL-related latent factor. The circular diagram displays the top enriched GO terms in three categories: Biological Process (BP), Cellular Component (CC), and Molecular Function (MF). The outer ring denotes the GO term IDs, with color coding representing ontology categories (blue: BP, red: CC, green: MF). The inner arcs illustrate the number of enriched genes and background genes, while the color intensity corresponds to the –log_10_(P value). Enriched GO terms were concentrated in neuronal development, synaptic organization, cell–cell interaction, and intracellular signaling processes. The circular plot summarizes biological process, cellular component, and molecular function categories.

GO enrichment analysis further characterized the biological functions of genes associated with the latent AL factor. Significant enrichment was observed across biological process (BP), cellular component (CC), and molecular function (MF) categories after multiple-testing correction (Supplementary Table 6, Fig. 5).

Within the BP category, enriched terms were predominantly related to neuronal development and cell–cell interaction, including dendrite development, regulation of neuron projection development, and cell junction assembly. These processes are essential for neural connectivity and structural plasticity, suggesting that genetic liability to AL may involve altered neurodevelopmental and synaptic organization.

Consistent with this, CC enrichment highlighted synapse-related components, such as glutamatergic synapse, postsynaptic density, and neuron-to-neuron synapse, indicating that synaptic structure and signaling are central cellular substrates underlying the shared genetic architecture of AL.

At the MF level, enriched terms included cadherin binding, protein tyrosine kinase activity, and phospholipid binding, reflecting involvement of cell adhesion molecules, intracellular signaling cascades, and membrane-associated regulatory processes.

### 3.7 Fine-mapping analysis

Fine-mapping analyses identified 51 high-confidence putative causal variants (mean posterior probability> 0.95) distributed across multiple biologically relevant genomic regions (Supplementary Table 7).

Several fine-mapped variants mapped to genes with established roles in metabolic regulation and energy homeostasis. For example, rs1800961 within HNF4A and rs42125 within MLXIPL, both key regulators of lipid and glucose metabolism, exhibited high posterior probabilities, reinforcing the contribution of metabolic pathways to AL. In addition, rs73048234 located near TOMM40 and variants within MYBPC3 and SH2B1 further suggest involvement of mitochondrial function, cardiometabolic regulation, and insulin signaling.

Notably, multiple high-confidence variants were mapped to genes implicated in neuronal signaling and synaptic function, including CADM3, SLC30A3, PDE4B, and PTPRD, consistent with enrichment of synaptic and neurodevelopmental pathways observed in the GO and KEGG analyses. Variants within immune- and stress-related genes, such as CRPP1, SIK3, and TRIM48, further highlight the multisystem nature of the genetic liability to AL.

### 3.8 Transcriptome-wide association study (TWAS)

**Fig. 6:**
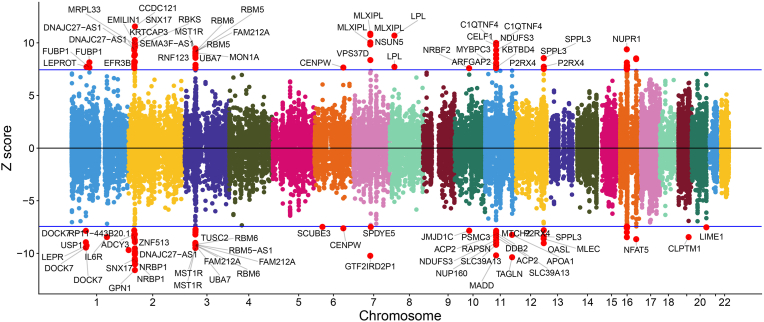
Manhattan plot of transcriptome-wide association study (TWAS) Z-scores. Each point represents a gene-based TWAS association across the genome, ordered by chromosomal position. The horizontal line indicates the genome-wide significance threshold (|Z| > 7.44), corresponding to P < 5 × 10^−14^.

We subsequently performed TWAS using the FUSION framework to identify gene-level expression signals associated with AL. Transcriptome-wide association analysis identified a total of 841 genes showing nominal evidence of association with the latent AL factor across relevant tissues. Applying stringent colocalization (PP4 > 0.8) and model performance (MODELCV.R^2^ > 0.02) thresholds yielded 159 high-confidence TWAS genes likely mediating GWAS associations with AL (Supplementary Table 8, Fig. 6).

These genes were primarily expressed in metabolic- and neuroendocrine-related tissues, suggesting their potential involvement in the biological stress response and energy homeostasis underlying the AL. Subsequently, fine-mapping analysis of the genomic structural equation model data was performed using FOCUS, which identified 327 genes as potentially causal signals associated with AL.

To further refine putative causal genes underlying AL, we integrated TWAS results with Bayesian fine-mapping using FOCUS and colocalization analysis. By applying stringent criteria requiring both strong GWAS–eQTL colocalization (COLOC PP4 > 0.8) and high posterior inclusion probability from FOCUS (pip >0.8), we identified 113 genes with convergent evidence of causality (Supplementary Table 9). For example, HNF4A, a key transcriptional regulator of metabolic homeostasis, and MLXIPL, a central mediator of glucose and lipid metabolism, showed strong support across TWAS, colocalization, and fine-mapping analyses. In addition, BDNF-related loci were retained in the TWAS–FOCUS intersection, consistent with the involvement of neurotrophic signaling in multisystem stress regulation.

### 3.9 GSEA identifies core pathways driving genetic susceptibility to AL

**Fig. 7:**
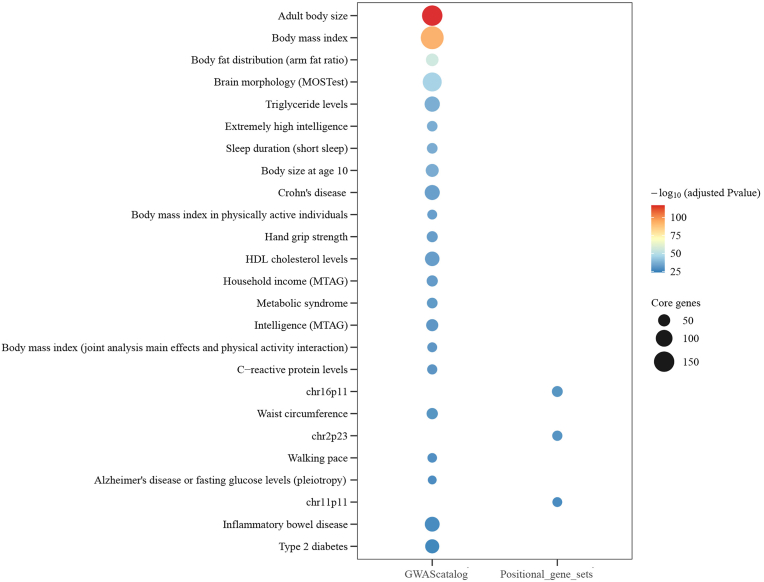
Functional gene-set enrichment of prioritized genes associated with the AL-related latent factor. The bubble plot illustrates the top 25 significantly enriched pathways identified by the functional enrichment analysis. Each bubble represents an enriched pathway or gene set. Bubble size indicates the number of core genes, while the color gradient corresponds to the enrichment significance (−log_10_ adjusted P value). The bubble plot shows the top enriched gene sets, with recurrent signals related to lipid metabolism, nutrient-stress sensing, neuronal regulation, and cellular stress pathways.

GSEA was used to profile pathway-level genetic signals underlying AL. Enrichment results were ranked by adjusted P value, and the most recurrent driver genes across highly ranked sets were considered pathway contributors. Dominant signals mapped to lipid metabolism, followed by curated modules involving nutrient-stress sensing and synaptic/neuronal regulation (Supplementary Table 10, Fig. 7).

The top-enriched pathways were related to lipid metabolism and lipoprotein processing, including triglyceride hydrolysis, cholesterol transport, and plasma lipoprotein assembly and clearance. Recurrent driver genes included LPL, APOA1, APOA4, APOA5, APOC3, APOE, ANGPTL4, NR1H3, and LIPC, consistent with prior evidence showing that cardiometabolic stress biology is strongly influenced by lipid regulatory networks.

A second enrichment cluster highlighted nutrient-stress sensing and energy regulation, implicating AMPK-related metabolic responses and insulin/glucose stress adaptation pathways. Curated gene sets within this group were driven by genes including MLXIPL, SH2B1, GCKR, and FTO, indicating that genetic variation influencing carbohydrate-lipid conversion and central energy balance regulation contributes to interindividual differences in cumulative stress burden.

Enriched neurobiological modules highlighted neuronal and synaptic regulation, involving BDNF, PTPRD, PTPRJ, CADM2, GRIA1, and NRXN family genes. These genes have documented roles in synaptic plasticity, stress adaptation, and neural circuit responsiveness to chronic stress exposure.

A regulatory-stress module showed enrichment for DNA damage repair, oxidative stress adaptation, autophagy-lysosomal regulation, and immune activation signatures. Driver genes included PDE4B, VPS18, TRAF3, RHOA, MADD, USP1, all previously reported in cellular stress and immune activation studies.

### 3.10 Cell-type–specific heritability enrichment

To investigate the cellular contexts through which genetic variation associated with AL may exert its effects, we performed cell type–specific heritability enrichment analyses using the CELLECT framework (Supplementary Table 11).

Using single-cell transcriptomic profiles from the Tabula Muris dataset, a significant enrichment signal was observed for non-myelinating neuronal cell populations (FDR <0.01), whereas no significant enrichment was detected for glial or immune-related cell types. Given the cross-species nature of this dataset, these results were interpreted as indicative of broad neuronal involvement rather than species-specific effects.

Consistent with this observation, analyses based on human transcriptomic resources revealed robust enrichment across multiple brain regions. Using GTEx expression data, significant signals were observed in cortical and subcortical structures, including the frontal cortex (BA9), anterior cingulate cortex (BA24), basal ganglia (putamen, caudate, nucleus accumbens), hippocampus, amygdala, hypothalamus, and cerebellum (FDR <0.05).

Further support was provided by chromatin-based enrichment analyses, which demonstrated strong heritability enrichment in active regulatory elements across several brain regions, particularly within the dorsolateral prefrontal cortex, cingulate gyrus, hippocampus, and temporal cortex, as marked by H3K27ac, H3K4me1, and H3K4me3 signals. Enrichment was also observed in fetal brain regulatory annotations, suggesting that part of the genetic architecture of AL may act through early neurodevelopmental regulatory mechanisms.

### 3.11 Partitioning of SNP heritability across genomic regions

Partitioned SNP heritability analysis was conducted using S-LDSC to evaluate the contribution of functional genomic categories to the total heritability of the latent allostatic factor (Supplementary Table 12).

This analysis revealed that heritability was disproportionately concentrated in evolutionarily conserved elements, which exhibited the strongest enrichment despite representing a small fraction of the genome. In addition, significant enrichment was observed across multiple classes of regulatory genomic features, including active histone modification marks, DNase I hypersensitive sites, and super-enhancer regions, indicating a prominent role for non-coding regulatory variation.

In contrast, repressed chromatin annotations showed enrichment estimates below unity, suggesting relative depletion of heritability in transcriptionally inactive regions. Coding regions, although comprising a limited proportion of SNPs, also demonstrated elevated enrichment, consistent with contributions from both coding and regulatory mechanisms.

Collectively, these findings indicate that the genetic architecture of AL is primarily shaped by variants residing in conserved and regulatory genomic elements, supporting a model in which non-coding functional variation plays a dominant role.

## 4 Discussion

In this study, we applied Genomic SEM to large-scale GWAS summary statistics to model AL as a latent genetic construct derived from cardiovascular, inflammatory, and metabolic biomarkers. AL reflects the cumulative physiological burden arising from chronic stress–induced dysregulation across interconnected biological systems, including cardiovascular, metabolic, immune, and neuroendocrine pathways [6]. In empirical research, AL is commonly operationalized as a composite index derived from biomarkers across several physiological domains rather than as a single directly observed trait. By applying Genomic SEM, we modeled the common genetic variance across five AL-related phenotypes and derived a latent genetic factor indexing coordinated variation across selected cardiovascular, inflammatory, and metabolic domains.

The factor-loading pattern further clarified the biological meaning of this latent construct (Supplementary Table 3). CRP showed the strongest standardized loading, followed by BMI and TG, whereas WBC showed a moderate loading and SBP showed a smaller contribution. This pattern suggests that the latent factor should not be interpreted as a uniformly weighted representation of all physiological systems involved in AL. Rather, it appears to capture an AL-related genetic dimension that is more strongly anchored in inflammatory and metabolic regulation, while still retaining contribution from cardiovascular variation.

The biological interpretation of these findings should be considered in light of the composition of the latent factor, as empirical AL indices are typically constructed from biomarker combinations across cardiovascular, metabolic, immune-inflammatory, and neuroendocrine systems, with substantial variation in indicator selection across studies [29]. Because the input indicators include metabolic, inflammatory, and cardiovascular biomarkers, enrichment of lipid metabolism, insulin signaling, inflammatory regulation, and cardiometabolic pathways is biologically expected and should not be interpreted as AL-specific. Rather, these results indicate that the shared genetic liability captured by the latent factor converges on biological systems that are central to the cardiometabolic and inflammatory dimensions of AL.

Although the SNP-based heritability of the latent AL factor was modest on the observed scale, this pattern is consistent with expectations for higher-order composite phenotypes derived from multiple correlated traits [13]. Importantly, LD score regression intercepts were close to unity, indicating that the observed inflation in test statistics was primarily attributable to polygenic signal rather than population stratification or confounding. These results support the validity of the latent AL construct and provide a robust foundation for downstream functional analyses. Nevertheless, the modest heritable signal warrants cautious interpretation. Downstream analyses, including functional annotation, fine-mapping, TWAS, pathway enrichment, and cell-type enrichment, should be viewed as prioritizing plausible genes, pathways, and regulatory contexts associated with the AL-related latent factor, rather than establishing definitive biological mechanisms.

The main contribution of this study is therefore conceptual as well as analytical. Previous genomic studies have extensively examined individual cardiometabolic, inflammatory, or metabolic traits, and recent multivariate genomic analyses have characterized shared genetic components of related disease or metabolic phenotypes [14]. In contrast, the present study uses AL as the organizing framework to model shared genetic liability across physiological systems that are commonly interpreted as downstream indicators of cumulative stress-related burden. This framing does not imply that the latent factor is specific to AL, but it provides a way to examine whether the shared genetic component across selected AL-related biomarkers maps onto biologically interpretable pathways, regulatory genomic regions, and tissue or cell-type contexts. Thus, the value of the model lies in connecting multivariate genetic architecture with the AL concept of coordinated physiological dysregulation.

Functional annotation of lead SNPs identified by the latent AL GWAS revealed that the majority of associated variants were located in non-coding regions, particularly intronic and intergenic regions. This distribution is consistent with observations across a wide range of complex traits, where disease-associated variants preferentially localize to regulatory elements rather than protein-coding sequences [30]. Partitioned heritability analyses further demonstrated significant enrichment of AL-associated heritability within conserved regions, promoters, enhancers, and active chromatin marks, including H3K27ac, H3K4me1, and H3K4me3. This pattern suggests that genetic susceptibility to AL is more likely mediated by regulatory effects on gene expression than by alterations in protein-coding sequences.

Pathway enrichment analyses converged on interconnected metabolic, endocrine, neurobiological, and immune-related pathways. KEGG and GSEA results emphasized insulin resistance, AMPK and PI3K–Akt signaling, lipid metabolism, and HPA-axis–related pathways, while GO analyses highlighted neuronal development, synaptic organization, and cell–cell adhesion processes. Collectively, pathway-level analyses point to the involvement of energy metabolism, neuroendocrine signaling, immune processes, and neural connectivity, indicating that AL-related genetic effects extend across multiple interconnected biological systems.

GSEA further reinforced these findings by demonstrating enrichment of lipid metabolism and lipoprotein-related gene sets, alongside neuronal and renal cell-type signatures. These convergent pathway-level results are consistent with the conceptualization of AL as a phenotype emerging from multisystem physiological dysregulation rather than isolated biological processes [31].

To refine genome-wide association signals and prioritize putative causal genes, we integrated Bayesian fine-mapping with TWAS and colocalization analyses. This approach reduced broad association signals to a limited set of high-confidence variants and genes, several of which converge on metabolic and neurobiological processes relevant to AL.

Among these candidates, HNF4A showed particularly strong support, with an exonic variant (rs1800961) exhibiting high posterior probability and predicted deleteriousness. The convergence of fine-mapping and transcriptome-based evidence supports HNF4A as a plausible candidate gene linking the latent AL-related factor to transcriptional regulation of metabolic processes. Consistent with this interpretation, HNF4A functions as a key regulator of hepatic glucose and lipid metabolism and has been repeatedly implicated in insulin resistance, dyslipidemia, and type 2 diabetes [32]. Its prioritization is consistent with the loading pattern of the latent factor and with enrichment of insulin resistance and metabolic signaling pathways observed in the KEGG and GSEA analyses.

A similar pattern was observed for MLXIPL, which was also prioritized through fine-mapping. Rather than reflecting a generic metabolic association, MLXIPL specifically links carbohydrate availability to lipid synthesis and energy storage. Genetic variation in MLXIPL has been associated with circulating triglyceride levels and lipid metabolic traits [33]. Given that TG and BMI contributed substantially to the latent factor, MLXIPL may primarily reflect the cardiometabolic component of the shared genetic architecture captured by the model.

Neurobiological candidates were also prominently represented. BDNF, prioritized through TWAS and colocalization analyses, is a key regulator of synaptic plasticity, neuronal survival, and stress-related neural adaptation. Extensive experimental and human studies have demonstrated that altered BDNF signaling mediates the effects of chronic stress on brain structure and function, and contributes to stress-related affective and cognitive phenotypes [34]. In the present study, BDNF-related signals should be interpreted as suggesting potential overlap between the AL-related latent factor and broader neurobiological regulatory processes.

Additional support for neuroendocrine integration was provided by SH2B1, an adaptor protein that modulates insulin and leptin receptor signaling. Genetic variants in SH2B1 have been associated with obesity, insulin resistance, and cardiometabolic risk [35]. Thus, SH2B1 may reflect shared genetic architecture between the AL-related latent factor and broader cardiometabolic or energy-balance pathways.

Beyond these focal genes, several other prioritized loci—including PDE4B, SLC30A3, and PTPRD—are involved in intracellular signaling and synaptic organization. Although discussed more briefly, their functional annotations are consistent with the enrichment of neuronal development and synaptic pathways observed in the GO and cell-type–specific analyses. Collectively, these gene-level findings provide convergent prioritization signals pointing to metabolic regulation, neuroendocrine-related signaling, and neural connectivity as biological processes relevant to the AL-related latent factor.

Cell-type–specific enrichment analyses pointed to the central nervous system as a primary biological context through which AL-associated genetic variation exerts its effects. Enrichment signals were observed in neuronal populations across multiple brain regions, including the prefrontal cortex, cingulate cortex, hippocampus, basal ganglia, and limbic structures. These regions are central to stress appraisal, emotional regulation, and neuroendocrine integration, suggesting that genetic susceptibility to AL may be preferentially mediated through neural circuits involved in stress processing. Such a pattern is consistent with prior evidence linking chronic stress exposure to alterations in corticolimbic structure and function.

Further support was provided by chromatin-based enrichment analyses, which demonstrated strong heritability enrichment in active regulatory elements across several brain regions, particularly within the dorsolateral prefrontal cortex, cingulate gyrus, hippocampus, and temporal cortex. Enrichment of fetal brain regulatory annotations suggests that part of the genetic architecture of AL may act through early neurodevelopmental regulatory mechanisms. This observation is consistent with the developmental origins of health and disease (DOHaD) framework, which posits that early-life regulatory processes shape long-term vulnerability to stress-related disorders [36].

Although some cell-type analyses relied on murine single-cell transcriptomic data, this approach is supported by substantial conservation of gene regulatory programs across mammalian species, particularly in fundamental neuronal and metabolic pathways. Moreover, convergence with human-based GTEx and chromatin datasets mitigates concerns regarding cross-species inference.

The present study benefits from a multilevel integrative design that combines multivariate genetic modeling with diverse functional genomics approaches, allowing triangulation of evidence across independent methods. However, several limitations warrant consideration. First, the latent AL construct in this study was defined by the available cardiovascular, inflammatory, and metabolic GWAS phenotypes. Although these domains are central to empirical AL indices, alternative operationalizations incorporating HPA-axis activity, autonomic function, glucose regulation, cholesterol fractions, or additional cytokine markers may yield partially different latent structures. Future studies with broader and more harmonized biomarker GWAS resources are needed to evaluate the robustness of AL genetic models across different indicator sets. Second, analyses were largely restricted to individuals of European ancestry, limiting generalizability to other populations. Third, genetic effects were modeled assuming shared architecture across sexes. Given established sex differences in immune, metabolic, and stress-response traits, sex-specific genetic architectures or gene–sex interactions may contribute to AL and were not explored in the current study. Finally, given the low observed-scale SNP heritability of the latent AL factor, gene-level and pathway-level findings should be interpreted as hypothesis-generating prioritization signals. Although integrative genomic analyses can highlight plausible candidate genes, pathways, and regulatory contexts, experimental and longitudinal studies are required to establish causal mechanisms.

Taken together, the novelty of this study is not limited to applying multiple post-GWAS tools to AL-related biomarkers. Rather, it lies in using Genomic SEM to translate the AL framework into a latent genetic model of shared cardiometabolic and inflammatory burden. This approach provides a system-level genetic perspective on AL-related physiology, while also highlighting important uncertainties. The latent factor was more strongly represented by inflammatory and metabolic indicators, its SNP-based heritability was modest, and downstream gene-level findings should be interpreted as prioritization signals. Future studies incorporating broader biomarker panels, environmental stress measures, longitudinal data, and multi-ancestry GWAS resources will be needed to determine how generalizable this genetic operationalization of AL is across populations and AL definitions.

## 5 Conclusion

In summary, this study delineates the polygenic architecture of AL by integrating Genomic SEM with fine-mapping, transcriptome-wide association analyses, pathway enrichment, and cell type–specific heritability profiling. By jointly modeling five AL–related component traits (SBP, WBC, CRP, BMI, and TG), we identified a latent genetic factor capturing the shared biological basis of multisystem stress regulation.

Across multiple analytic layers, our results consistently implicate metabolic and neuroendocrine regulatory pathways as central drivers of genetic susceptibility to AL. Integrative fine-mapping and TWAS prioritized plausible candidate genes related to metabolic regulation, including HNF4A and MLXIPL, highlighting the role of glucose and lipid metabolism, insulin resistance, and energy homeostasis. In parallel, genes involved in neuronal signaling and plasticity, such as BDNF, SH2B1, PDE4B, and PTPRD, underscore the contribution of central nervous system regulation to cumulative stress burden.

Functional enrichment analyses further reinforced this framework, revealing convergent signals in lipid metabolism, insulin signaling, synaptic organization, and neurodevelopmental pathways. Cell type–specific and chromatin-based analyses localized these genetic effects predominantly to stress-relevant brain regions, including the prefrontal cortex, hippocampus, cingulate cortex, and basal ganglia, with additional evidence for regulatory activity in developmentally active chromatin states.

Collectively, these findings position AL as a genetically regulated metabolic–neurobiological integrative phenotype, shaped by coordinated perturbations in energy metabolism and central stress-processing circuits. This work refines genome-wide association signals to biologically interpretable genes and pathways, providing a focused molecular framework for future mechanistic studies of chronic stress–related health outcomes.

## Data availability statement

The GWAS summary statistics used in this study are publicly available. Data from the IEU GWAS database can be accessed at https://gwas.mrcieu.ac.uk/. Detailed accession numbers for each dataset are provided in the Methods section.

## Funding statement

This study was supported by the Key Scientific Research Project of Higher Education Institutions in Henan Province (24B320034).

## CRediT authorship contribution statement

**Songhao Chai:** Data curation, Formal analysis, Visualization. **Wenjun Jin:** Writing – original draft. **Zhenghao Cui:** Investigation, Validation. **Bo Li:** Writing – original draft, Writing – review & editing.

## Declaration of competing interest

The authors declare that they have no known competing financial interests or personal relationships that could have appeared to influence the work reported in this paper.

## Supplementary Information

The supplementary materials associated with this article include:

Supplementary Tables 1–12: Detailed statistical results, additional phenotype descriptions, heritability estimates, model fit indices, and genetic correlation matrices referenced in the manuscript.

Supplementary Data File (AL_GWAS.zip): Contains raw input files used in the structural genomic modeling, including GWAS summary statistics.

All supplementary materials are provided as separate files and should be consulted alongside the main text for a complete understanding of the methodology and findings.
