# Supplementary figures and images for: Genomic structural equation modeling elucidates the genetic mechanisms underlying allostatic load
Source: Compr Psychoneuroendocrinol. 2026 Jun 25;27:100357. doi: 10.1016/j.cpnec.2026.100357 (PMC13329553; doi:10.1016/j.cpnec.2026.100357)

Observed  $-\log_{10}(\rho)$

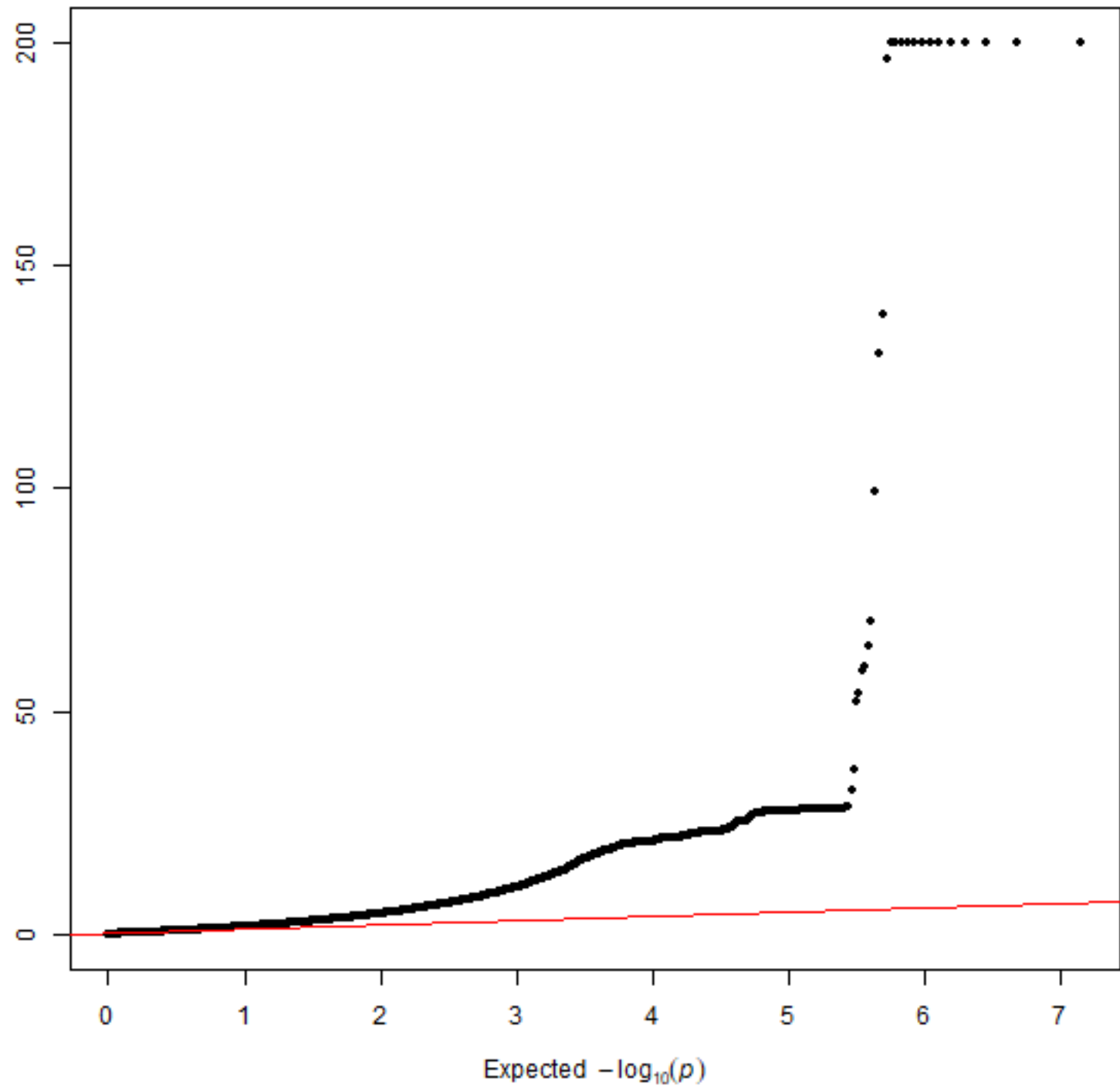

Supplement: Multimedia component 13 [file mmc13.pdf]
